# Macrolide Therapy in Respiratory Viral Infections

**DOI:** 10.1155/2012/649570

**Published:** 2012-06-06

**Authors:** Jin-Young Min, Yong Ju Jang

**Affiliations:** Department of Otolaryngology, Asan Medical Center, University of Ulsan College of Medicine, 388-1 Pungnap-2dong, Songpa-gu, Seoul 138-736, Republic of Korea

## Abstract

*Background*. Macrolides have received considerable attention for their anti-inflammatory and immunomodulatory actions beyond the antibacterial effect. These two properties may ensure some efficacy in a wide spectrum of respiratory viral infections. We aimed to summarize the properties of macrolides and their efficacy in a range of respiratory viral infection. *Methods*. A search of electronic journal articles through PubMed was performed using combinations of the following keywords including macrolides and respiratory viral infection. *Results*. Both *in vitro* and *in vivo* studies have provided evidence of their efficacy in respiratory viral infections including rhinovirus (RV), respiratory syncytial virus (RSV), and influenza virus. Much data showed that macrolides reduced viral titers of RV ICAM-1, which is the receptor for RV, and RV infection-induced cytokines including IL-1**β**, IL-6, IL-8, and TNF-**α**. Macrolides also reduced the release of proinflammatory cytokines which were induced by RSV infection, viral titers, RNA of RSV replication, and the susceptibility to RSV infection partly through the reduced expression of activated RhoA which is an RSV receptor. Similar effects of macrolides on the influenza virus infection and augmentation of the IL-12 by macrolides which is essential in reducing virus yield were revealed. *Conclusion*. This paper provides an overview on the properties of macrolides and their efficacy in various respiratory diseases.

## 1. Introduction

Macrolides are a group of antibiotics whose activity stems from the presence of the macrolide ring to which one or more deoxy sugars, usually cladinose and desosamine, may be attached. Lactone rings are usually 14, 15 or 16 membered. Macrolides which tend to accumulate within leukocytes and are transported into the site of infection are used to treat respiratory and soft-tissue infections caused by Gram-positive bacteria such as *Streptococcus pneumoniae* and *Haemophilus influenzae*. In addition to the typical antibiotic effect, two properties including the anti-inflammatory and the immunomodulatory actions are inherent in this group of drugs. These anti-inflammatory and immunomodulatory actions of macrolides encouraged a number of researchers to explore a potential application of macrolides even for respiratory viral infection [1–5].

The purpose of this paper is to summarize the properties of macrolides and their efficacy in a range of respiratory viral infection.

## 2. Search Strategy

We performed an electronic article search through PubMed using combinations of the following keywords: macrolides (azithromycin, clarithromycin, dirithromycin, erythromycin, roxithromycin, and telithromycin) and respiratory viral infection (respiratory syncytial virus, rhinovirus, adenovirus, metapneumovirus, influenza virus, and parainfluenza virus). All types of articles such as randomized controlled trials, clinical observational cohort studies, review articles, and case reports were included.

## 3. Anti-Inflammatory and Immune Modulation Effects of Macrolides

At present, macrolides are known to possess anti-inflammatory and immunomodulatory actions extending beyond their antibacterial activity in pulmonary inflammatory disorders such as diffuse panbronchiolitis (DPB), asthma, and cystic fibrosis. Both *in vitro* and *in vivo* data show macrolides to downregulate prolonged inflammatory response, reduce airway mucus secretion, inhibit the bacterial adhesion biofilm, reduce the production of reactive oxygen species, inhibit neutrophil activation and mobilization with an acceleration of the apoptotic process, and also block the activation of nuclear transcription factors [6–11]. After macrolides accumulating within cells, they may interact with receptors or second messengers responsible for the regulation of cell cycle and cellular immunity.

However, the anti-inflammatory effects observed with macrolides are modest if compared to the anti-inflammatory effects of corticosteroids and require much higher doses, questioning their real use as an anti-inflammatory agent. Further studies are needed.

## 4. Macrolides and Respiratory Viral Infections

As macrolides have anti-inflammatory and immunomodulatory effect, the scenario thus depicted is sufficiently suggestive to consider the possible use of these drugs in respiratory viral infection presenting an inflammatory basis. The common causes of respiratory viral infection include rhinovirus (RV), respiratory syncytial virus (RSV), adenovirus, metapneumovirus, influenza virus, and parainfluenza virus. Recent studies have shown that the high mortality rate of respiratory virus infections is a result of an overactive inflammatory response. Respiratory viral infections are characterized by the appearance of cytokine storms which is extreme production and secretion of numerous proinflammatory cytokines. Severity of infection is closely related with virus-induced cytokine dysregulation which is responsible for the development of fatal clinical symptoms, such as massive pulmonary edema, acute bronchopneumonia, alveolar hemorrhage, reactive hemophagocytosis, and acute respiratory distress syndrome. Numerous *in vitro*, *in vivo,* and clinical studies have established that viruses are potent inducers of various cytokines and chemokines including TNF-*α*, interferon (IFN)-*γ*, IFN-*α*/*β*, IL-6, IL-1, MIP (macrophage inflammatory protein)-1, MIG (monokine induced by IFN-*γ*), IP (interferon-gamma-inducible protein)-10, MCP (monocyte chemoattractant protein)-1, RANTES, and IL-8 [12–17].

It is known that macrolides downregulate the inflammatory cascade, they attenuate excessive cytokine production in viral infections, and they may reduce virus-related exacerbation. Furthermore, macrolides may influence phagocyte activity by modifying their miscellaneous functions including chemotaxis, phagocytosis, oxidative burst, bacterial killing, and cytokine production [18]. It has also been reported that macrolides could interfere with the influenza virus replication cycle, resulting in the inhibition of virus production from infected cells, mainly by inhibiting intracellular hemagglutinin HA0 proteolysis [19, 20]. There are still controversies in the effects of macrolides in respiratory viral infections. The following review will introduce recent research findings regarding the effectiveness of macrolides antibiotic on different forms of respiratory viral infections (Table 1).

### 4.1. Cell Culture Studies

Among *in vitro*, *in vivo*, and clinical studies, *in vitro* studies, especially cell culture studies, were most frequently performed to evaluate the effect of macrolides on respiratory viral infection. Numerous *in vitro* studies with various respiratory virus revealed that macrolides are effective on respiratory viral infections.

RV is the most common cause of viral upper respiratory tract infections (URIs) and is responsible for about one half of all cases of the common cold. Although RV does not cause necrosis of epithelial cells or substantial histological changes in nasal mucosa, RV infection induces the hypersecretion of mucus, as well as the increased expression and secretion of various cytokines, including interleukin (IL)-6, IL-8, IL-9, IL-1b, IL-11, and TNF-*α*, and the influx of neutrophils, which correlate with the severity of cold symptoms [35, 36]. It is well known that approximately 90% of more than 100 different RV serotypes bind to ICAM-1, and RV infection upregulates ICAM-1 expression on airway epithelial cells, thus facilitating further viral attachment and entry [36, 37]. As ICAM-1 is the receptor for the major RV and since IL-1b, IL-6, and IL-8 play significant roles in the pathophysiology of RV infection, macrolides which are known to have inhibitory effect on those cytokines may be able to modulate inflammatory processes during RV infection. Studies have been done to determine anti-inflammatory properties of macrolide antibiotics against RV infection.

Among these macrolides, erythromycin is the first drug which was studied about their efficacy on RV. Erythromycin is a macrolide antibiotic with potent anti-inflammatory effects that is used for treating chronic lower respiratory tract infections. Suzuki et al. examined the effects of erythromycin on RV (RV2 and RV14) infection in airway epithelium [23]. In their study, erythromycin reduced the supernatant RV14 titers, RV14 RNA, the susceptibility to RV14 infection, and the production of ICAM-1 and cytokines which was upregulated by RV14. Erythromycin also reduced the supernatant RV2 titers, RV2 RNA, the susceptibility to RV2 infection, and cytokine production, although the inhibitory effects of erythromycin on the expression of the low-density lipoprotein receptor, the minor RV receptor, were small. In addition, erythromycin may also modulate airway inflammation by reducing the production of proinflammatory cytokines and ICAM-1 induced by RV infection. Erythromycin reduced the NF-*κ*B activation by RV14 and decreased the number of acidic endosomes in the epithelial cells.

Another type of macrolide antibiotics, bafilomycin A1 also inhibits infection of RV, in human airway epithelial cells by the reduction of ICAM-1 and by affecting the acidification of endosomes, where RV RNA enters into the cytoplasm of infected cells [22]. Bafilomycin A1 and erythromycin could reduce proinflammatory cytokines including IL-6 after RV infection in airway epithelial cells [22, 38].

Jang et al. investigated the effect of clarithromycin on RV infection in A549 cells [24]. In their study, clarithromycin treatment inhibited the RV-induced increase in ICAM-1 mRNA and protein, as well as the RV induced secretion of IL-1*β*, IL-6, and IL-8. These effects were greater in cells treated with 10 *μ*M than in those treated with 100 *μ*M CM, and the maximum effect was observed 3 days after viral infection. In contrast, secretion of IL-8 was not inhibited significantly when clarithromycin was added at the time of viral infection. In their study, RV titer, as measured by culture on MRC-5 cells, was reduced by clarithromycin, with the degree of reduction being greater when clarithromycin was added 3 days before infection than it was added at the time of infection. Through these findings, they suggested that, in A549 cells, clarithromycin inhibits the induction of ICAM-1 expression, cytokine elaboration, and viral infection.

Secondary bacterial infection by respiratory viral infection is important pathogenic mechanism in rhinosinusitis. Wang et al. investigated the inhibitory effects of clarithromycin on secondary bacterial infection after RV infection [26]. RV-induced URIs may enhance secondary bacterial infections via upregulation of cell adhesion molecules in the nasal mucosa, leading to acute bacterial rhinosinusitis. *Staphylococcus aureus* binds to human fibronectin (Fn) and *Haemophilus influenza* adheres to the carcinoembryonic antigen-related cell adhesion molecules (CEACAMs) of epithelial cells. In their study, clarithromycin treatment alone had no effect on the baseline levels of mRNA and protein expression of Fn and CEACAM, but significantly reduced the RV-induced increases in the mRNA and protein levels of Fn and CEACAM to the levels found in noninfected controls. They also demonstrated clarithromycin treatment-induced reduction of bacterial adhesion to RV-infected human nasal epithelial cells. Thus, they suggested that clarithromycin may be effective at preventing secondary acute bacterial RS following RV infection.

Several macrolide antibiotics are reported to inhibit airway mucus hypersecretion induced by several stimuli. The main component of mucus is mucin. MUC5AC and MUC5B are reported to constitute 95–98% of secreted mucin in airways. Mucus with a high concentration of MUC5AC or MUC5B has a high viscosity and is likely to cause airway narrowing. Erythromycin attenuated RV14-induced MUC5AC production and secretion in cultured human tracheal epithelial cells [25]. MUC5AC mRNA expression was also attenuated by erythromycin treatment, suggesting that erythromycin affects pretranscriptional mechanisms. Furthermore, erythromycin attenuated RV14-induced p44/42 MAPK activation.

Gielen et al. investigated the anti-RV (RV 1B and RV16) potential of macrolides including azithromycin, erythromycin, and telithromycin, through the induction of antiviral gene mRNA and protein [27]. Azithromycin, but not erythromycin or telithromycin, significantly increased RV 1B- and RV 16-induced IFNs and IFN-stimulated gene mRNA expression and protein production. Furthermore, azithromycin significantly reduced RV replication and release. RV-induced IL-6 and IL-8 protein and mRNA expressions were not significantly reduced by azithromycin before treatment. These results demonstrated that azithromycin has antirhinoviral activity in bronchial epithelial cells by increasing the production of IFN-stimulated genes.

In addition, the duration of macrolide therapy could affect the immune response. *Ex-vivo* studies seem to indicate that short-term administration of macrolides may enhance the immune response, whereas long-term administration results in immunosuppression [39].

RSV bronchiolitis is the most common lower respiratory tract infection in infancy, occurring in 90% of children of 2 yrs or under. Development of an effective therapy against the short-term morbidity by RSV bronchiolitis could be important in reducing subsequent morbidity. RSV causes widespread damage to bronchial epithelium and stimulates epithelial cells to secrete a wide range of pro-inflammatory cytokines and chemokines. IL-8 is a key chemokine produced by RSV-infected airway cells and is involved in the activation and recruitment of neutrophils. Neutrophils play a major role in the pathophysiology of RSV bronchiolitis.

Several reports showed that macrolide antibiotics may also modulate airway inflammation induced by RSV infection [28–30]. Suppressive effects of macrolides on the plasma IL-4, IL-8, and eotaxin levels may have a role in suppression of airway hyperresponsiveness or may inhibit cholinergic neuroeffector transmission in human airway smooth muscle, thereby influencing bronchial tone [31, 39–43]. Macrolides attenuate the release of eotaxin, granulocyte-macrophage colony-stimulating factor (GMCSF), and RANTES. It may also protect epithelial cells at inflamed sites by inhibiting the release of reactive oxygen species from eosinophils [32, 44].

In the RSV infection, RhoA, isoform A of the Ras-homologus (Rho) family, has various functions including stimulus-evoked cell adhesion and motility, enhancement of contractile response, and cytokine production. The activated form of RhoA moves to the cell membrane and is implicated in the RSV infection [30, 45, 46]. Asada et al. reported that bafilomycin A1 and clarithromycin inhibit infection by RSV and decrease the susceptibility of cultured human tracheal epithelial cells to RSV infection, partly through the reduced expression of activated RhoA which is an RSV F protein receptor [30]. Because activated RhoA interacts with the RSV F protein, these findings suggest that clarithromycin may inhibit RSV infection, partly through the reduction of activated RhoA in the cells. Clarithromycin also reduced baseline and RSV infection-induced release of proinflammatory cytokines in supernatant fluids including IL-1*β*, IL-6, and IL-8 [30]. It has been shown that viral titers in supernatant fluids and RNA of RSV in the human tracheal epithelial cells increased with time, and clarithromycin reduced viral titers of RSV in supernatant fluids concentration-dependently, RNA of RSV replication, and the susceptibility to RSV infection.

Influenza virus is another common cause of respiratory viral infection. Human influenza virus infection causes rapid onset constitutional symptoms, including fever and lower respiratory tract symptoms, and also induces exacerbations of bronchial asthma and chronic obstructive pulmonary disease (COPD) in the winter. Human influenza viruses attach to sialic acid with an *α*2,6linkage (SA*α*2,6Gal) on the airway epithelial cells. The viruses are then delivered into the cytoplasm, and ribonucleoproteins (RNPs) of viruses, which include viral RNA, are released from acidic endosomes into the cytoplasm of the cells. There are several reports which showed the efficacy of macrolide antibiotics on influenza virus infection. Miyamoto et al. showed the ability of clarithromycin in inhibition of human influenza A virus production *in vitro *at a middle-to-late stage of viral replication cycle [20]. They found that treatment with clarithromycin at a final concentration of 25 *μ*g/mL had a strong inhibitory effect on plaque reduction of the tested human influenza A viruses. In addition to decrease of progeny virus production, clarithromycin decreased apoptotic cell numbers of infected host cells. These findings suggested that clarithromycin acts directly on virus-infected cells and contributes to the prevention of virus production by inhibiting viral replication in infected host cells. The influenza virus replication cycle can be divided into 5 steps: (1) binding of viral hemagglutinin to sialic acid receptor on host cell surface (adsorption step), (2) internalization of virus by receptor-mediated endocytosis and fusion of viral HA2 with endosomal membranes triggered by influx of protons through M2 channel (endocytosis and fusion step), (3) release of viral genes into the cytoplasm (uncoating step), (4) packaging of viral proteins with viral genes after viral RNA replication, transcription and translation, and budding of new viruses (packaging and budding step), and (5) release of new viruses by sialidase cleaving sialic acid receptors (release step) [20]. Clarithromycin had no or little inhibitory effect on hemagglutination, hemolysis activity (membrane fusion), and sialidase activity. These results suggest that decrease of progeny virus production is not due to inhibition of viral hemagglutinin and sialidase activities, which play an important role at the beginning and the end of viral replication, respectively. After clarithromycin was incubated with virus-infected cells at different times, it has been found that clarithromycin predominantly inhibited viral replication after viral adsorption to host cells at about the 4–7th hour [20]. Clarithromycin therefore might act on middle-to-late stage of viral replication cycle, presumably *via *blockage of producing viral protein. These findings strongly encourage the potential use of clarithromycin as an anti-influenza virus chemotherapeutic agent.

### 4.2. Animal Studies

Compare to *in vitro* studies, *in vivo* studies were relatively rare. Further *in vivo* animal studies are needed with various respiratory viruses.

There were several reports which evaluated the effects of macrolide on influenza-virus-induced respiratory infection. Sato et al. evaluated the effects of erythromycin on influenza-virus-induced pneumonia in mice infected with a lethal dose of influenza virus A/Kumamoto/Y5/67 (H2N2) [31]. In their report, erythromycin may have substantial therapeutic value for various acute inflammatory disorders such as influenza-virus-induced pneumonia. The effects were by inhibiting inflammatory cell responses and suppressing nitric oxide (NO) which plays critical role in the pathologic events of various inflammatory diseases, overproduced in the lung. Regarding the NO, erythromycin treatment resulted in a dose-dependent decrease in the level of nitrite/nitrate (metabolites of NO) in the serum and the NO-synthase-(NOS-) inducting potential in the lungs of the virus-infected mice. As a result, administration of erythromycin significantly improved the survival rate of mice infected with influenza virus, and the survival rate of the virus-infected mice increased in a dose-dependent fashion. It has also been found in their study that the induction of IFN-*γ* in the mouse lung was inhibited and the number of inflammatory cells after virus infection was significantly reduced by erythromycin treatment on day 6 after infection.

In addition to being an antibiotic able to prevent complications and aggravation of symptoms, clarithromycin has been reported to alleviate pneumonia secondary to influenza virus infection in mice [32]. In their study, clarithromycin has been shown to suppress the inflammatory cytokines such as TNF-*α*, but augment IL-12 production, resulting in alleviation of influenza infection itself in infected mice [32]. These studies indicated that clarithromycin may play a role *in vivo *as an immunomodulator for influenza virus infection.

The protective role of IL-12 against influenza infection was assessed by analyzing the efficacies of orally administered clarithromycin as an immunomodulator and intranasal administration of recombinant IL-12 in influenza-virus-infected mice. Tsurita et al. reported that, in infected mice, clarithromycin at 20 mg/mouse/day significantly elevated the levels of IL-12 and IFN-*γ* in the bronchoalveolar lavage on days 2 and 3, respectively, but the levels in the sera were not affected [32]. In accordance with the locally elevated level of f IL-12, clarithromycin reduced virus yield and the number of infiltrated cells, the severity of pneumonia, and mortality of the treated mice. Thus, the augmentation of IL-12 production in the respiratory tract was essential in reducing virus yield in the early phase of influenza and may be crucial for recovery from influenza infection [32].

There is another report which revealed the effect of macrolides on reducing the receptor for virus on the airway epithelial cells and reducing entry of virus into the cytoplasm. Human seasonal influenza viruses and classical H1N1 swine influenza viruses bind to SA*α*2,6Gal, and most avian and equine viruses bind to SA*α*2,3Gal [47]. Clarithromycin reduced the expression of SA*α*2,6Gal, a receptor for human influenza, on the mucosal surface of human tracheae, and reduced the number of acidic endosomes from which viral RNPs enter into the cytoplasm. These findings suggest that a clinically used clarithromycin may inhibit type A seasonal human influenza virus infection via reducing its receptor on the airway epithelial cells and reducing entry of viral RNPs, into the cytoplasm. Although the mechanisms for the reduction of SA*α*2,6Gal expression by clarithromycin are uncertain, these effects are similar to those of clarithromycin on the reduced expression of activated RhoA, one of receptors for RSV, and on inhibition of RSV infection [30]. These effects are also similar to those of erythromycin on the reduced expression of ICAM-1, a receptor for RV, and on inhibition of the RV infection.

Recently, Yamaya et al. demonstrated that clarithromycin reduces FluA viral titers and cytokines secretion in supernatant fluids and susceptibility of the cells to infection by the virus [34].

### 4.3. Clinical Studies

Although numerous *in vivo* studies have established that macrolides have inhibitory effects on respiratory viral infections, the outcomes of clinical studies are controversial and the clinical benefits of macrolides in respiratory virus infection are still uncertain.

In* in vitro* study, Jang et al. reported that clarithromycin inhibits the RV-induced induction of ICAM-1 expression, cytokine elaboration, and viral infection in A549 cells [24]. However, there is a controversial report performed in a double-blinded clinical trial showing that clarithromycin treatment had little or no effect on the severity of cold symptoms or the intensity of neutrophilic nasal inflammation [21]. The discrepancy between the results of *in vitro* study by Jang et al. and those of the *in vivo* clinical trial may be due to differences in dosage or mode of treatment. For example, in the clinical trial, 1,000 mg·day^−1^ of clarithromycin, a higher dose than the 250 mg·day^−1^ usually used for low-dose, long-term treatment [48], was started 24 h before inoculation of RV. However, it was found that clarithromycin started 3 days before RV infection was more effective than clarithromycin started at the time of infection and that 10 *μ*M clarithromycin, the usual blood level in clinical use, was more effective than 100 *μ*M in reducing viral titer and cytokine secretion.

In addition, there are controversies about the effective duration of macrolide therapy. *Ex vivo *studies seem to indicate that short-term administration of macrolides may enhance the immune response, whereas long-term administration results in immunosuppression [39]. However, other study described that short-term administration of a macrolide is not beneficial for acute uncomplicated colds caused by RV infection [21].

Severe RSV infections during early infancy are associated with the excessive production of Th2 cytokines, which has been suggested as a risk factor for the later development of asthma and allergic sensitization [49]. Macrolides may normalize the Th1/Th2 lymphocyte balance [50]. They regulate immunologic activities by enhancing production of IFN-*γ* and by reducing production of IL-4 and IL-5. Treatment that restores the Th1/Th2 cytokine balance to the relative type 1 predominance may ameliorate short- and long-term effects of RSV disease. Tahan et al. studied the use of 3 weeks of macrolide therapy in the treatment of RSV bronchiolitis in a double-blind, randomized, placebo-controlled trial [28]. In their study, treatment with clarithromycin daily for 3 weeks was associated with a statistically significant reduction in the length of hospital stay, the duration of need for supplemental oxygen, the need for *β*2-agonist treatment, and readmission to the hospital within 6 months after discharge. Furthermore, there were significant decreases in plasma IL-4, IL-8, and eotaxin levels after 3 weeks of treatment with clarithromycin. As previously described, RSV is the leading cause of viral lower respiratory tract disease (LRTD) in infants and young children. Nearly half of all hospitalized infants with RSV LRTD are treated with antibiotics. In contrast to favorable effects of macrolides on RSV infection reported in number of papers, Kneyber et al., however, reported that the use of macrolide antibiotics would not lead to a reduced duration of hospitalization in mild-to-moderate RSV LRTD [29]. In their study, azithromycin was not associated with a stronger resolution of clinical symptoms represented by the RSV symptom score.

Various inflammatory mediators are suggested to be associated with the pathogenesis and severity of influenza virus infection [42]. Increases in proinflammatory cytokines and monokines, including interleukin IL-1, IL-6, and IL-8, are observed in the serum in the patients and in the lung of mice infected with influenza virus [41, 42]. Although the clinical benefits of macrolides in influenza virus infection are still uncertain, reduction of proinflammatory cytokines by clarithromycin may modulate influenza-virus-induced inflammation and severity of the disease and may prevent COPD exacerbations. Clarithromycin inhibits the activation of NF-*κ*B, migration of neutrophils, and the production of proinflammatory cytokines by interfering with extracellular signal-regulated kinases [39]. It also promotes the induction of sIgA and IgG in the airway fluids of mice infected with influenza A virus [51]. Sawabuchi et al. investigated the immunomodulatory effects of clarithromycin on mucosal immune responses in the nasopharyngeal aspiration of pediatric patients with influenza [33]. In their study, low induction of antiviral sIgA which represents the first immunological barrier to pathogens was observed in the oseltamivir, an antiviral neuraminidase inhibitor, treatment group. However, the addition of clarithromycin to oseltamivir augmented sIgA production and restored local mucosal sIgA levels, indicating that clarithromycin boosted the nasopharyngeal mucosal immune response in children presenting with influenza A, even in those treated with oseltamivir who had low production of mucosal anti-viral sIgA [33].

## 5. Conclusions

Macrolides possess anti-inflammatory and immunomodulatory properties extending beyond their antibacterial activity. They downregulate the inflammatory cascade, attenuate excessive cytokine production in viral infections, and they may reduce virus-related exacerbations. Based on existing evidence, macrolides may be considered as promising treatment option in treatment of respiratory viral infections. However, confirmation in larger series, as well as identification of their precise mechanism affecting virus-induced inflammation or viral replicationn, is still awaited.

## Figures and Tables

**Table 1:** Macrolide studies evaluating efficacy on various respiratory viral infection.

Virus	Study	Macrolide	Dose	Method	Parameter	Results	Ref
*Rhinovirus *							
RV16	Abisheganaden et al. (2000)	Clarithromycin	500 mg	Clinical trial	Symptom, nasal peak flow, weight of nasal secretion, cytokines (IL-6, IL-8 in nasal lavage fluid)	No effect	[21]
RV14	Suzuki et al. (2001)	Bafilomycin A1	0.1 *μ*M	*In vitro* (human tracheal epithelial cells)	RV titer, ICAM-1, cytokines (IL-1*β*, IL-6, IL-8, TNF-*α*)	Inhibition	[22]
RV14RV2	Suzuki et al. (2002)	Erythromycin	10 *μ*M	*In vitro* (human tracheal epithelial cells)	RV titer, ICAM-1, cytokines (IL-1*β*, IL-6, IL-8, TNF-*α*)	Inhibition	[23]
RV16	Jang et al. (2006)	Clarithromycin	1, 10, 100 *μ*M	*In vitro* (A549 cells)	RV titer, ICAM-1, cytokines (IL-1*β*, IL-6, IL-8)	Inhibition	[24]
RV14	Inoue et al. (2008)	Erythromycin	10 *μ*M	*In vitro* (human tracheal epithelial cells)	MUC5AC	inhibition	[25]
RV16	Wang et al. (2010)	Clarithromycin	10 *μ*M	*In vitro* (Nasal epithelial cell)	Fn, CEACAM, bacterial adhesion (*S. aureus*, *H. influenza*)	Inhibition	[26]
RV16RV1b	Gielen et al. (2010)	Azithromycin, Erythromycin, Telithromycin	10 *μ*M	*In vitro* (Human bronchial epithelial cells)	mRNA of antiviral genes, type I IFN-*β*, type III IFN-*λ*1, IFN-*λ*2/3, IFN-stimulated genes, cytokines (IL-6, IL-8), RV replication, RV release	Azithromycin: inhibition, erythromycin, telithromycin:no effect	[27]
*Respiratory syncytial virus*							
RSV	Tahan et al. (2007)	Clarithromycin	15 mg/kg	Clinical trial	Cytokines (IL-4, IL-8, eotaxin, IFN-*γ*) duration of hospitalization, duration of need for supplemental oxygen, *β*2-agonist	Effective	[28]
RSV	Kneyber et al. (2008)	Azithromycin	10 mg/kg	Clinical trial	duration of hospitalization, duration of oxygen supplementation and nasogastric tube feeding, RSV symptom score, number of PICU referrals number of patients who received additional antibiotic treatment	No effect	[29]
RSV	Asada et al. (2009)	Bafilomycin A1 Clarithromycin	10 *μ*M	*In vitro* (human tracheal epithelial cells)	Viral titers, cytokines (IL-1*β*, IL-6, IL-8)	Inhibition	[30]
*Influenza virus*							
A/Kumamoto/Y5/67	Sato et al. (1998)	Erythromycin	1.0 or 3.3 mg/kg	*In vivo* (Mice)	Survival rate, body weight, cytokines (IFN-*γ*, TNF-*α*, IL-1*β*), NO_2_^−^ /NO_3_^−^	Inhibition	[31]
A/PR/8/34 (H1N1)	Tsurita et al. (2001)	Clarithromycin	20 mg	*In vivo* (Mice)	Virus yield, severity of pneumonia, cytokines (IL-4, 6, 10, 12)	Inhibition IL-12: elevation	[32]
A/PR/8/34 (H1N1) A/Aichi/2/68 (H3N2) A/Memphis/1/71 (H3N2) A/WSN/33 (H1N1)	Miyamoto et al. (2008)	Clarithromycin	25 mg/mL	*In vitro* (MDCK cells, human lung epithelial A549 cells)	Multiple infection assay	Inhibition (middle to late stage of the viral replication cycle)	[20]
influenza A (H1N1) and (H3N2)	Sawabuchi et al. (2009)	Clarithromycin	5 mg/kg	Clinical trial	Antiviral sIgA, numbers of viral RNA copies, symptom	Inhibition	[33]
type A influenza virus (H3N2)	Yamaya et al. (2010)	Clarithromycin	10 *μ*M	*In vitro* (human tracheal epithelial cells)	Viral titer, cytokines (IL-1*β*, IL-6), viral RNA	Inhibition	[34]

RV*:* rhinovirus, IL*:* interleukin, ICAM-1*:* intercellular adhesion molecule-1, TNF*-α*: tumour necrosis factor alpha, Fn*:* fibronectin, CEACAM*:* carcinoembryonic antigen-related cell adhesion molecules, IFN*:* interferon, RSV*:* respiratory syncytial virus, and MDCK*:* Mardin-Darby canine kidney.
